# Long Range Regulation of Human *FXN* Gene Expression

**DOI:** 10.1371/journal.pone.0022001

**Published:** 2011-07-08

**Authors:** Novita Puspasari, Simone M. Rowley, Lavinia Gordon, Paul J. Lockhart, Panos A. Ioannou, Martin B. Delatycki, Joseph P. Sarsero

**Affiliations:** 1 Bruce Lefroy Centre for Genetic Health Research, Murdoch Childrens Research Institute, Royal Children's Hospital, Parkville, Victoria, Australia; 2 Cell and Gene Therapy, Murdoch Childrens Research Institute, Royal Children's Hospital, Parkville, Victoria, Australia; 3 Bioinformatics Unit, Murdoch Childrens Research Institute, Royal Children's Hospital, Parkville, Victoria, Australia; 4 Department of Paediatrics, The University of Melbourne, Royal Children's Hospital, Parkville, Victoria, Australia; 5 Department of Clinical Genetics, Austin Health, Heidelberg, Victoria, Australia; Texas A&M University, United States of America

## Abstract

**Background:**

Friedreich ataxia (FRDA) is the most common form of hereditary ataxia characterized by the presence of a GAA trinucleotide repeat expansion within the first intron of the *FXN* gene. The expansion inhibits *FXN* gene expression resulting in an insufficiency of frataxin protein.

**Methodology/Principal Finding:**

In this study, computational analyses were performed on the 21.3 kb region upstream of exon 1 of the human *FXN* gene and orthologs from other species in order to identify conserved non-coding DNA sequences with potential regulatory functions. The conserved non-coding regions identified were individually analyzed in two complementing assay systems, a conventional luciferase reporter system and a novel Bacterial Artificial Chromosome (BAC)-based genomic reporter. The BAC system allows the evaluation of gene expression to be made in the context of its entire genomic locus and preserves the normal location and spacing of many regulatory elements which may be positioned over large distances from the initiation codon of the gene.

**Conclusions/Significance:**

The two approaches were used to identify a region of 17 bp located approximately 4.9 kb upstream of the first exon of the *FXN* gene that plays an important role in *FXN* gene expression. Modulation of *FXN* gene expression was found to be mediated by the action of the Oct-1 transcription factor at this site. A better understanding of *cis-*acting regulatory elements that control *FXN* gene expression has the potential to develop new strategies for the upregulation of the *FXN* gene as a therapy for FRDA.

## Introduction

Friedreich ataxia (FRDA) is an autosomal recessive disorder characterized by neurodegeneration and cardiomyopathy. It is the most common form of hereditary ataxia with an estimated 2–3 affected individuals per 100,000 in European populations [1] and an estimated carrier frequency of 1 in 110 [2]–[4]. The causative gene, *FXN*, is located on the long arm of chromosome 9 (9q13-21.1). The *FXN* gene encodes the mitochondrial protein frataxin, which plays an important role in iron-sulfur cluster biogenesis [5], [6]. Homozygosity for a GAA trinucleotide repeat expansion within the first intron of the *FXN* gene is the most common cause of FRDA. Normal alleles contain 6–34 uninterrupted GAA repeats. The majority of individuals with FRDA have between 67 to over 1,300 GAA repeats in both *FXN* alleles. The non-translated GAA repeat expansion results in inhibition of gene expression and an insufficiency of frataxin.

An inverse correlation exists between the size of the smaller expanded allele and transcript levels, the amount of residual frataxin produced and the age of onset of disease symptoms. Heterozygous carriers of a GAA repeat expansion produce about half the normal level of frataxin and are asymptomatic. As the GAA repeat expansion mutation does not alter the coding sequence of the gene, it is hypothesized that any increase in frataxin levels should prove beneficial, while a several-fold increase could be sufficient to halt disease progression.

There is currently limited information on the regulation of the *FXN* gene. The 1,255 bp region upstream of the *FXN* coding region contains the minimal promoter. The region is rich in repetitive elements which appear to be important in promoter activity. A TATA box is not apparent and Inr/DPE-like elements found in the vicinity of the transcription start site are not required for gene expression [7]. A putative E-box and Mt binding site within the first intron were shown to contribute to *FXN* promoter activity [8]. Transcription factors SRF and TFAP2 have been shown to bind sequences in the promoter and to enhance *FXN* expression [9]. There is evidence that the GAA repeat expansion generates a heterochromatin-mediated gene silencing effect [10], [11]. Changes in both DNA methylation and histone modification have been described [8], [10], [12]–[15]. The mechanism by which the GAA expansion results in epigenetic changes in the *FXN* gene is not known. A recent report hypothesizes that CTCF depletion and antisense transcription in the 5′UTR of the *FXN* gene is associated with epigenetic silencing [16]. There is currently no data on the position of any long-range, *cis*-acting regulatory sequences or of associated transcription factors that may control *FXN* gene expression.

A better understanding of how *cis-*acting, long-range regulatory control elements surrounding the human *FXN* gene, and the *trans-*acting protein factors that act upon them, are responsible for gene expression in healthy and affected individuals, has the potential to lead to the development of new strategies that target the upregulation of the *FXN* gene as a therapy for FRDA [17]. We performed comparative genomic analysis of the 21.3 kb region upstream of the first exon of the human *FXN* gene and 16 orthologs from other species to identify conserved non-coding DNA sequences with potential regulatory functions. The conserved non-coding regions were individually analyzed in two assay systems, a conventional small plasmid luciferase reporter system and a novel Bacterial Artificial Chromosome (BAC)-based genomic reporter. The latter system permits the evaluation of gene expression to be made in the context of its entire genomic locus and preserves the normal location and spacing of many regulatory elements that may be positioned over large distances in the surrounding chromosomal region. We have identified a minimal region of 17 bp, located approximately 4.9 kb upstream of the first exon of the *FXN* gene that plays an important role in normal *FXN* gene expression. Computational methods identified a number of putative transcription factor sites within this sequence, of which the Oct-1 transcription factor was found to influence *FXN* gene expression via this site.

## Results

### Bioinformatic identification of conserved non-coding DNA sequences upstream of the *FXN* gene

**Figure 1:**
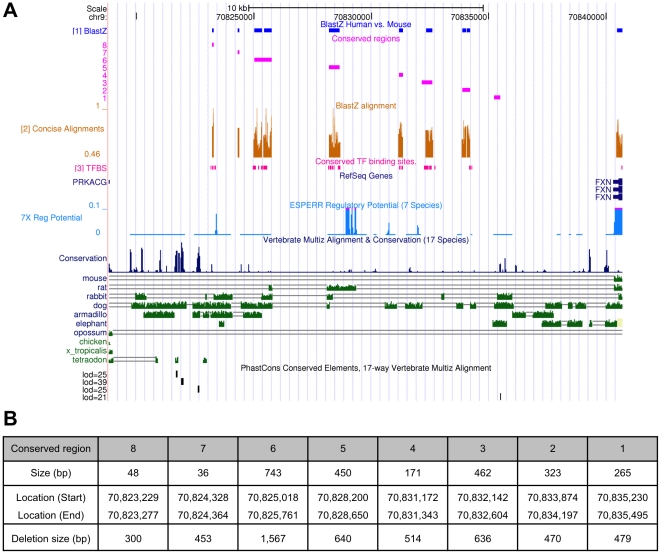
Cross species sequence comparison. Analysis of cross species sequence comparison of the upstream non-coding region of the *FXN* gene utilizing data from the UCSC Genome Browser using the March 2006 (NCBI36/hg18) assembly (http://genome.cse.ucsc.edu). (A) The position of the human *FXN* gene on chromosome 9 is shown. The BLASTZ track shows the BLASTZ alignment of human *FXN* and the mouse *Fxn* genes. BlastZ alignments were taken from GenomeTraFaC [27]. The genomic sequences with flanking (upstream to 5′ and downstream to 3′) 40 kb base pairs of more than 12,000 human and mouse orthologous gene pairs that had a validated RefSeq ID from the Reference Sequence data of NCBI were downloaded from the UCSC Genome Browser (Human May 2004 and March 2006 assemblies, and Mouse August 2005 and February 2006 assemblies). Sequence alignment was done using the BlastZ algorithm of PipMaker. The location of each conserved non-coding region identified upstream of the human *FXN* gene is shown. The 7× regulatory potential (ESPERR Regulatory Potential) displays the regulatory potential (RP) scores computed from alignment of human, chimpanzee, macaque, mouse, rat, dog, and cow. The peaks illustrate the position of sequences conserved between the human *FXN* gene and orthologs in each of the individual species. The PhastCons track shows predictions of conserved elements produced by the PhastCons program, which identifies conserved elements given a multiple alignment and a hidden Markov model [20]. Both the ESPERR and PhastCons tracks were obtained from the UCSC Genome Browser [18]. (B) The location (UCSC Genome Browser, Human March 2006 assembly) and size of eight highly conserved regions located upstream of exon 1 of the *FXN* gene are shown. The size of the deletion encompassing each conserved region made in the RP11-265B8::Ex5a-EK-DsAmp (Dual Reporter) construct is indicated.

The *FXN* gene is located on the long arm of chromosome 9. Our particular focus was on the region upstream of the *FXN* gene coding region, specifically the 21.3 kb intergenic region between the *FXN* and *PRKACG* genes. Identification of regions that may contain potential long-range, *cis*-acting regulatory elements controlling the expression of the human *FXN* gene was achieved by examining comparative genomic and regulatory data available on the UCSC Genome Browser [18], [19] (Fig. 1A). Comparative genomic data that was utilized consisted of conserved elements predicted from multiple alignments by a phylogenetic hidden Markov model [20]–[23]. Regulatory data utilized consisted of regulatory potential (RP) scores computed from alignments of human, chimpanzee, macaque, mouse, rat, dog and cow [24], [25]. The RP score compares frequencies of short alignment patterns between known regulatory elements and neutral DNA (24,25). RP scores represent a mixture of conservation, composition and short pattern structure information gathered from alignments to suggest the position of new putative regulatory elements. RP scores have been shown to effectively discriminate regulatory from neutral DNA stretches [26].

The region upstream of *FXN* was further investigated for the presence of conserved regulatory elements including transcription factor binding sites [27]. Eight highly conserved regions were identified upstream of exon 1 of the *FXN* gene (Fig. 1B). The conserved regions are within a size range of 36 bp to 743 bp and each harbors homology of 40% to greater than 70% among the species, and is surrounded by otherwise divergent sequences (data not shown).

### Construction and characterization of a dual reporter bacterial artificial chromosome

Key control regions can be located large distances from the genes that they regulate and the normal location and spacing of many regulatory elements is crucial to facilitate physiological gene expression patterns. Genomic reporters, in which a reporter fusion is made to a gene in the context of its entire genomic locus on a Bacterial Artificial Chromosome (BAC) clone, preserve the positional relationships of regulatory elements in the surrounding chromosomal region, and facilitate the recapitulation of normal gene expression patterns.

We previously identified a 188 kb BAC clone (RP11-265B8) containing exons 1–5b of the human *FXN* locus and extensive flanking regions [28]. The genomic insert is able to successfully complement the embryonic lethal phenotype of homozygous *Fxn* knockout mice, indicating that key regulatory elements required for normal expression of the *FXN* gene are present within this clone [29]. Homologous recombination was used to introduce an EGFP-Kan/Neo cassette in-frame immediately following the last codon of exon 5a of the *FXN* gene (designated RP11-265B8::Ex5aEK) [28]. This construct encodes a frataxin-EGFP fusion protein that is expressed via the endogenous *FXN* promoter and under the control of regulatory elements surrounding the intact *FXN* locus.

The RP11-265B8::Ex5a-EK clone can only be maintained in bacterial cells but is suitable for transient transfection studies in eukaryotic cells. In regulatory studies utilizing small reporter constructs, a second reporter plasmid is typically co-transfected as a transfection control. Due to their large size, the transfection efficiency of BAC clones into mammalian cells is usually very low (around 1–10% depending on the size of the BAC clone and the cell line being transfected). Co-transfection of a small reporter plasmid would not be an accurate reflection of the BAC transfection efficiency, while co-transfection of a second BAC with a different reporter would prove technically challenging. To overcome this limitation, a second, independently expressed DsRed-Express reporter was introduced into the backbone of the RP11-265B8::Ex5a-EK clone. The chloramphenicol-resistance gene was replaced by the DsRed-Express reporter and an ampicillin-resistance gene. DsRed-Express has distinct emission spectra from EGFP and has been optimized for use in mammalian cells. The independent expression of the DsRed-Express gene serves as an internal control for transfection efficiency and vector copy number. Two fluorescent markers allows simultaneous monitoring of the effects of deleting putative *FXN* regulatory elements and the correction for any differences in transfection efficiencies. The integrity of the new RP11-265B8::Ex5a-EK-DsAmp (Dual Reporter) construct was confirmed by PCR and restriction enzyme digestion. The expression of EGFP and DsRed-Express was confirmed in the transient transfection assay in BHK21 cells (data not shown).

### Construction and characterization of BAC dual reporter deletion constructs

Each of the eight identified conserved non-coding regions was individually deleted in the context of the RP11-265B8::Ex5a-EK-DsAmp (Dual Reporter) construct. Each conserved region was substituted with a chloramphenicol-resistance gene flanked by FRT sites via homologous recombination. The chloramphenicol-resistance gene was subsequently removed of using Flp recombinase to leave a single FRT site in place of the conserved region. It was not always possible to design homology arms of the targeting cassette to precisely delete all of the conserved sequences due to the presence of repetitive elements or lack of unique sequences immediately surrounding some conserved regions. In such cases, some additional flanking sequence was also removed (Fig. 1B). The integrity of the required constructs was verified by assessing chloramphenicol-sensitivity and by PCR and restriction digest analyses.

In order to investigate the effect of the deletion of each conserved non-coding region on *FXN* gene expression, each construct was analyzed in transient transfection assays in a mammalian cell line. We have previously shown that the BHK-21 (baby hamster kidney) cell line is readily transfectable with large DNA constructs and suitable for the *in vivo* characterization of *FXN* gene expression [28].

**Figure 2:**
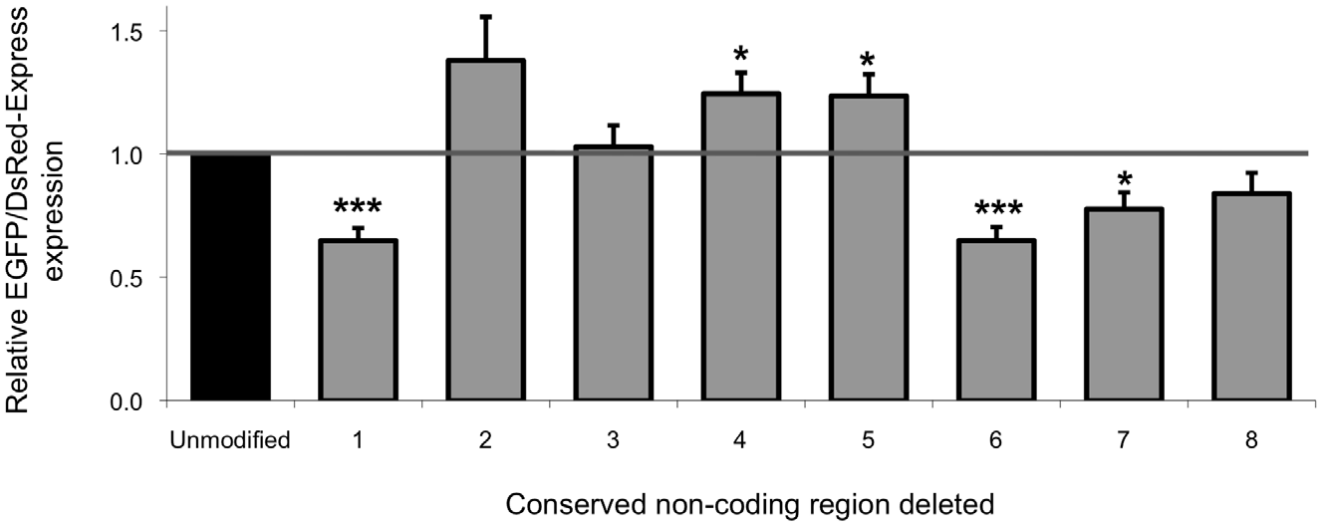
Expression analysis of BAC dual-reporter deletion constructs. BHK-21 cells were transfected with the RP11-265B8::Ex5a-EK-DsAmp (Dual Reporter) construct and derivatives individually containing deletions of the identified conserved non-coding regions. EGFP and DsRed-Express expression were measured by flow cytometry 72 hours post transfection. The expression of the different deletion constructs was compared to the expression of the control RP11-265B8::Ex5a-EK-DsAmp (Dual Reporter) construct. Assays were performed in triplicate on at least three independent occasions. Error bars represent standard error of the mean. *p<0.05, ***p<0.001 in comparison to the unmodified control RP11-265B8::Ex5a-EK-DsAmp (Dual Reporter) construct.

The EGFP/DsRed-Express ratio produced by the BAC containing a deletion of conserved region 1 was significantly lower (*p* = 0.0001) than the unmodified control RP11-265B8::Ex5a-EK-DsAmp (Dual Reporter) construct (Fig. 2). The deletion of conserved region 6 also resulted in significantly lower *FXN* gene expression (*p* = 0.0004). The deletion of conserved region 7 caused a reduction in expression (*p* = 0.01), but not to the same extent as deletion of conserved regions 1 and 6. In contrast, the removal of conserved regions 4 and 5 resulted in higher *FXN* expression levels (*p* = 0.02 and *p* = 0.03, respectively).

EGFP and DsRed-Express expression from each of the BAC dual-reporter deletion constructs was also analyzed by fluorescence microscopy. Supporting the flow cytometry data, a clear reduction in EGFP expression was observed in cells transfected with the BAC clones containing a deletion of either conserved region 1 or 6 (Fig. S1). Deletion of conserved region 7 also demonstrated lower EGFP expression although not to the same extent as in the constructs containing deletions of conserved regions 1 and 6 (Fig. S1).

### Analysis of the effects of upstream conserved non-coding regions on the *FXN* promoter

**Figure 3:**
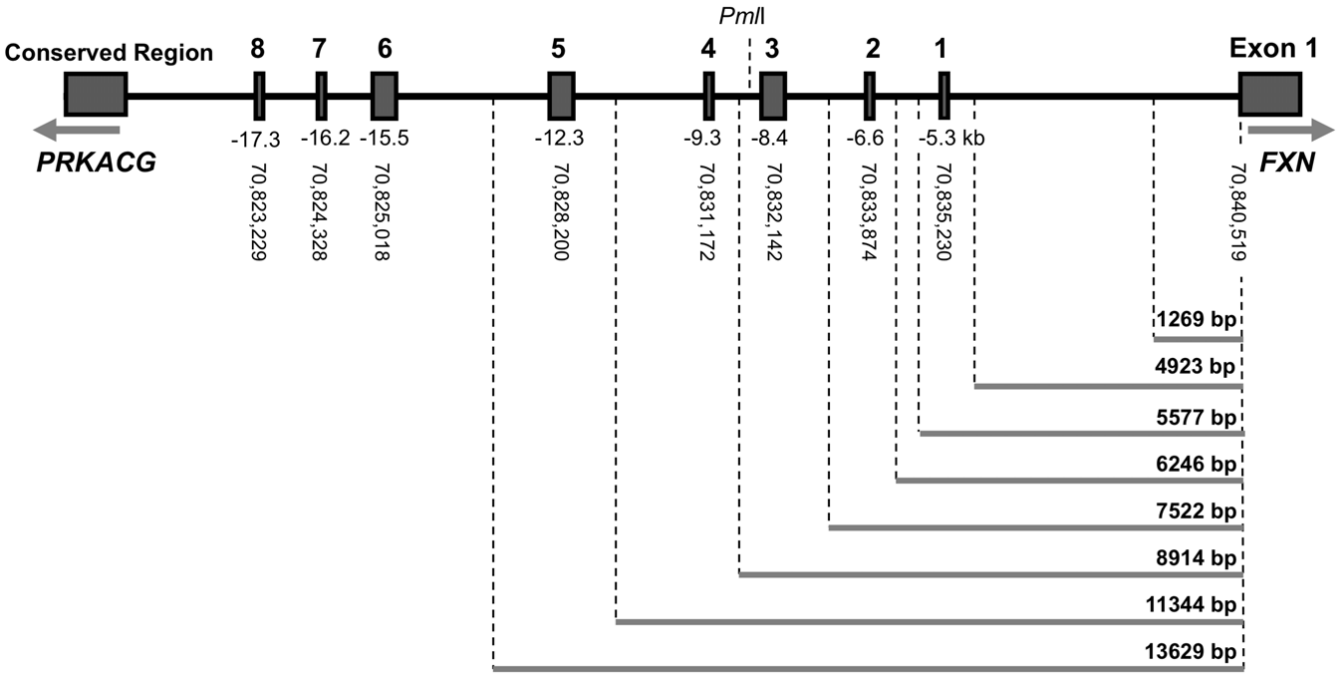
DNA fragments utilized in expression analyses. Schematic representation of PCR-amplified fragments containing the *FXN* promoter and conserved regions cloned into the pGL3-Basic vector. The size of each fragment (bp) and the inclusion of particular conserved regions are shown. The location of each of the identified conserved non-coding regions, their distance (kb) from exon 1 of the *FXN* gene and their coordinates (UCSC Genome Browser, Human March 2006 assembly) are indicated. (Not to scale.)

To complement the data obtained using the genomic reporter assay, the examination of *FXN* gene regulatory mechanisms was also assessed using small plasmid luciferase reporter constructs. A set of progressively longer, DNA fragments containing the endogenous human *FXN* promoter and one or multiple conserved non-coding regions was amplified by PCR and cloned into the pGL3-Basic vector (Fig. 3).

The set of pGL3-Basic derivative constructs containing the *FXN* promoter and one or multiple conserved non-coding regions were separately transfected into two mammalian cell lines, HeLa (cervical cancer) and BE(2)-M17 (neuroblastoma).

**Figure 4:**
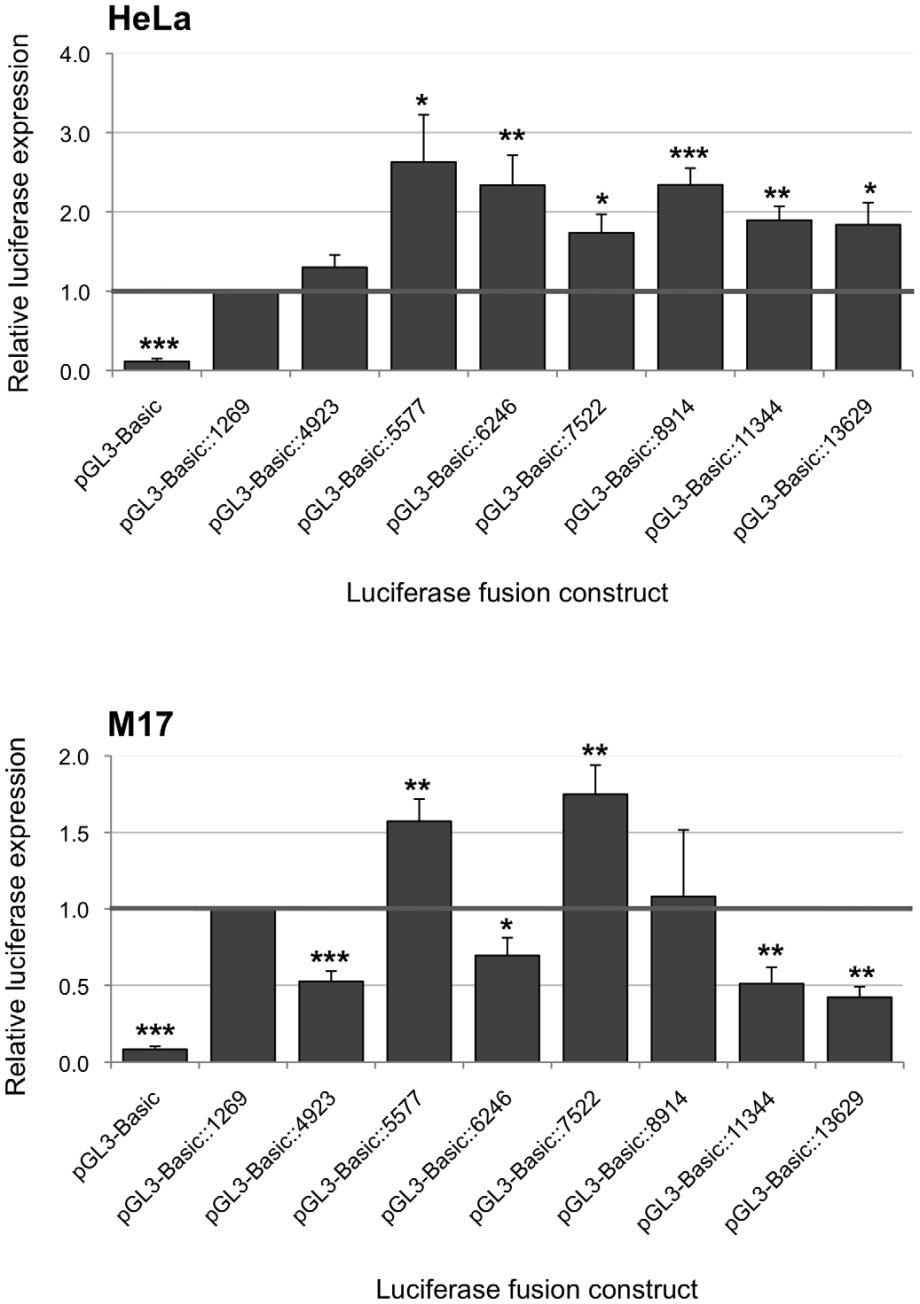
Regulatory effects of upstream conserved regions on the expression of the *FXN* promoter. DNA fragments containing the *FXN* promoter and one or multiple upstream conserved regions were inserted into the pGL3-Basic vector. The nomenclature of the vectors is based on the size of the cloned DNA fragments (bp) comprised of the sequence upstream of exon 1 of the *FXN* gene. Luciferase expression was assayed in HeLa and BE(2)-M17 cells. The overall levels of expression were compared to the pGL3-Basic::1269 vector, the expression value of which was set as 1. Assays were performed in triplicate on at least three independent occasions. Error bars represent standard error of the mean. *p<0.05; **p<0.01; ***p<0.001 in comparison to the control pGL3-Basic::1269 vector.

In both cell lines, the construct containing the shortest insert (1,269 bp region immediately upstream of exon 1 of the *FXN* gene) elicited much higher levels of luciferase expression than the unmodified pGL3-Basic vector without an insert (Fig. 4). These results support the findings of Greene et. al. [7] that this region contains the minimal *FXN* promoter. In the HeLa cell line, the next longest insert containing 4,923 bp of DNA but excluding any of the identified conserved regions, produced a similar level of expression. All of the other constructs, which contained an insert longer than 5,577 bp, produced a statistically significant increase in expression compared to that with the shortest insert. The greatest level of induction (over 2.5-fold) was seen with the construct that contained the 5,577 bp insert, which includes only conserved region 1. This result indicates that the presence of conserved region 1 plays a role in inducing the expression of the *FXN* gene, and which was only modestly modified in the presence of the other conserved regions. In the BE(2)-M17 cell line, a greater variation was observed in the effects on *FXN* gene expression depending on the conserved regions present in each construct (Fig. 4). Increased or reduced expression was observed depending on the length of the insert and presumably the presence of particular conserved regions. This could possibly be attributed to interactions between positive and negative regulatory roles of the different conserved regions in this particular cell line. Nonetheless, in accordance with the findings in the HeLa cell line, the construct that contained the 5,577 bp insert, which includes only conserved region 1, led to increased expression in the BE(2)-M17 cell line, albeit to a lesser extent than in HeLa cells.

As these results indicated that conserved region 1 harbors an important regulatory element that influences the expression of the *FXN* gene, subsequent experiments focused on refining the specific sequence(s) within this region responsible for the observed effects.

### Location of a regulatory element within conserved region 1

In order to delineate the location of a regulatory element within conserved region 1, a series of truncated constructs was generated. The initial focus was on the region located between 4,923 and 5,577 bp upstream of exon 1 of the *FXN* gene. Three PCR products were amplified with 5′ ends terminating at 5,023, 5,123 and 5,443 bp upstream of exon 1.

The three new constructs (designated pGL3-Basic::5023, pGL3-Basic::5123 and pGL3-Basic::5443) were transfected into HeLa and BE(2)-M17 cell lines. For this series of experiments, the normalized expression of the pGL3-Basic derivative constructs was compared to the construct containing the 5,577 bp fragment (pGL3-Basic::5577).

**Figure 5:**
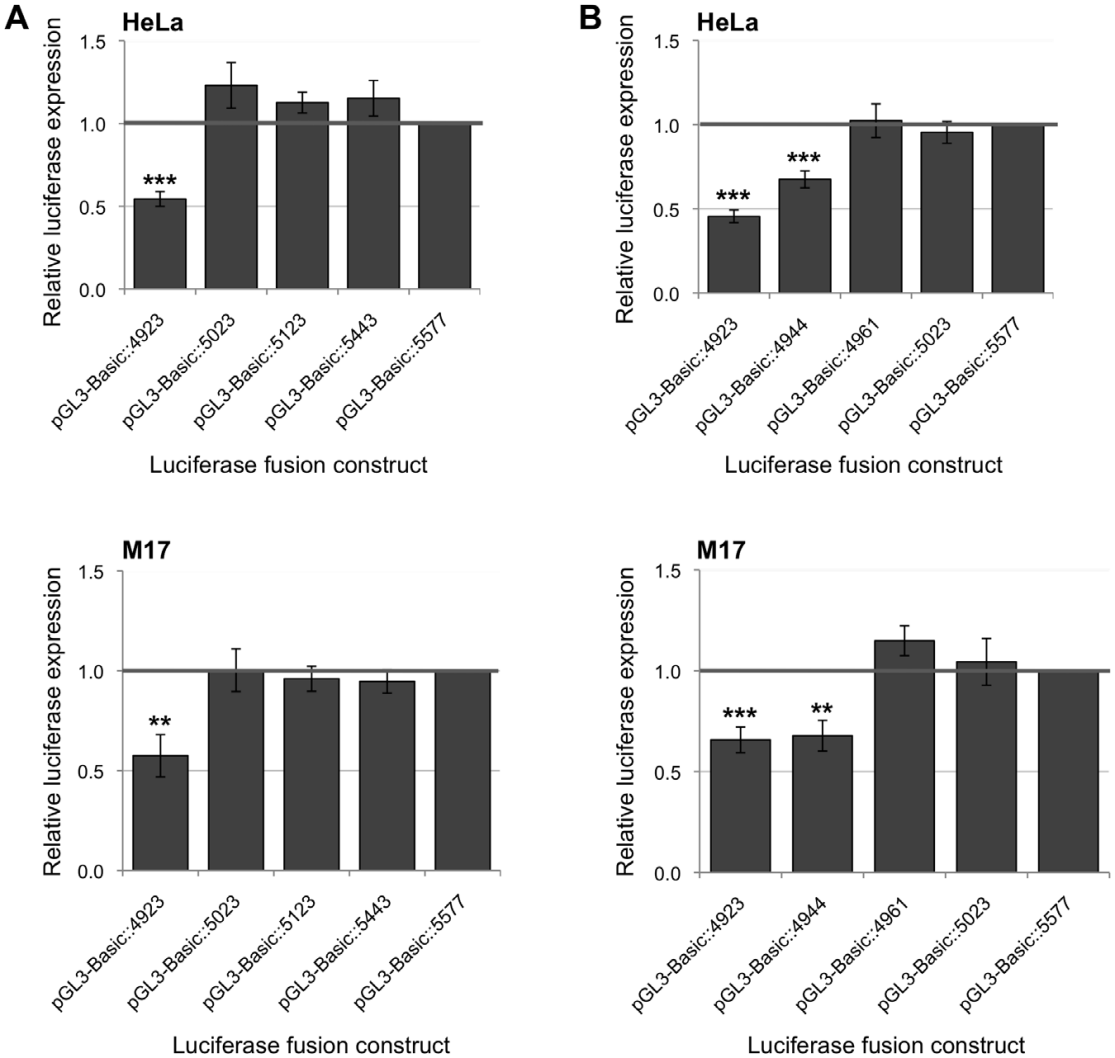
Regulatory effects of conserved region 1 on the expression of the *FXN* promoter. DNA fragments containing the *FXN* promoter and segments of conserved region 1 were inserted into the pGL3-Basic vector and luciferase expression was assayed in HeLa and BE(2)-M17 cells. The overall levels of expression were compared to the pGL3-Basic::5577 vector, the expression value of which was set as 1. (A) Fragments terminating 5,023–5,443 bp upstream of *FXN* exon 1; (B) Fragments terminating 4,944–5,023 bp upstream of *FXN* exon 1. Assays were performed in triplicate on at least three independent occasions. Error bars represent standard error of the mean. *p<0.05; **p<0.01; ***p<0.001 in comparison to the control pGL3-Basic::5577 vector.

The patterns of expression of all the constructs were very similar in both the HeLa and BE(2)-M17 cell lines. The new constructs containing all or part of conserved region 1 (pGL3-Basic::5023, pGL3-Basic::5123 and pGL3-Basic::5443) as well as the reference construct pGL3-Basic::5577 (which contains the entire conserved region 1 sequence) all elicited a significant increase in *FXN* gene expression in comparison to the pGL3-Basic::4923 construct (which excludes conserved region 1) (Fig. 5A). These results indicated that the regulatory element is located in the 100 bp region between 4,923 and 5,023 bp upstream of exon 1.

To further delineate the location of the regulatory element within conserved region 1, two further truncated constructs were generated terminating within the 100 bp region between 4,923 bp and 5,023 bp upstream of exon 1 of the *FXN* gene. These were designated pGL3-Basic::4944 and pGL3-Basic::4961. The levels of expression of both of the constructs were similar in the HeLa and BE(2)-M17 cell lines. There was no significant difference between the levels of expression elicited by the pGL3-Basic::5577 and the pGL3-Basic::4961 constructs (Fig. 5B). However, there was a clear reduction in expression produced by the pGL3-Basic::4944 construct; which was similar to the level of expression of the pGL3-Basic::4923 construct. These results indicated that a regulatory element is located in a 17 bp sequence (5′ CAAATTGCATTCCAGAGTGT 3′) between 4,944 bp and 4,961 bp upstream of exon 1 of the *FXN* gene.

The 17-bp region identified via the luciferase reporter assays was analyzed further in the RP11-265B8::Ex5a-EK-DsAmp (Dual Reporter) assay. The 17-bp sequence located between 4,944 bp and 4,961 bp upstream of exon 1 of the *FXN* gene was deleted by homologous recombination in the dual reporter BAC construct. The construct was analyzed in transient transfection assays in the BHK-21 cell line. The expression of EGFP from this deletion construct was compared to the unmodified control BAC construct.

**Figure 6:**
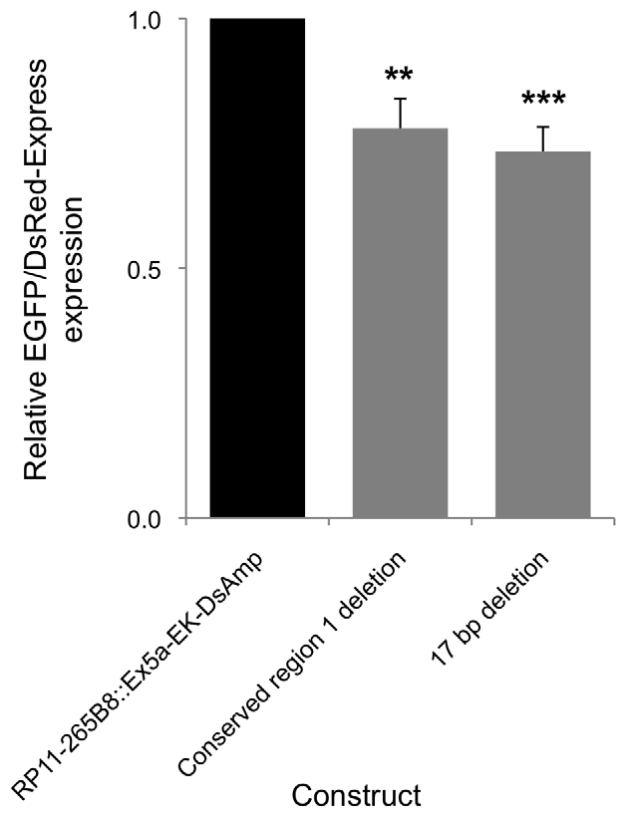
Expression analysis of BAC dual-reporter constructs containing deletions in conserved region 1. BHK-21 cells were transfected with the RP11-265B8::Ex5a-EK-DsAmp (Dual Reporter) construct and derivatives individually containing deletions of the entire conserved region 1 or the identified 17 bp sequence within conserved region 1. EGFP and DsRed-Express expression were measured by flow cytometry 72 hours post transfection. The expression of the different deletion constructs was compared to the expression of the control plasmid RP11-265B8::Ex5a-EK-DsAmp (Dual Reporter) construct. Assays were performed in triplicate on at least three independent occasions. Error bars represent standard error of the mean. **p<0.01, ***p<0.001 in comparison to the unmodified control RP11-265B8::Ex5a-EK-DsAmp (Dual Reporter) construct.

The level of *FXN* gene expression produced by the BAC construct containing the 17 bp deletion was significantly lower (*p* = 0.00007) than the unmodified control BAC. The level of expression was comparable to that produced by the BAC clone containing the entire deletion on the conserved region 1 (*p* = 0.0001) (Fig. 6). This was further confirmed by fluorescent microscope observation which clearly showed a reduction in EGFP expression in cells transfected with the BAC construct containing the 17 bp deletion compared to the control BAC, and comparable to the BAC clone containing the entire deletion on the conserved region 1 (Fig. S2). This result clearly indicated that the 17 bp region within conserved region 1 plays an important regulatory role in *FXN* gene expression.

### The identification of an Oct-1 transcription factor binding site within the 17 bp region of conserved region 1

The Alibaba 2.1, Match 1.0 and MatInspector programs were utilized to identify candidate regulatory elements present within the 17 bp sequence located between 4,944 bp and 4,961 bp upstream of exon 1 of the *FXN* gene. The Alibaba 2.1 program was utilized at two stringency parameters. At the lower stringency settings, four potential transcription factor binding sites were identified. These were binding sites for Octamer binding protein (Oct-1), Nuclear Factor 1 (NF-1), CCAAT/enhancer binding protein (C/EBPbeta) and Heat Shock Transcription Factor 1 (HSF-1). At the higher stringency setting, only the Oct-1 transcription factor binding site was identified. Using the Match 1.0 program, four putative transcription factor binding sites were identified; Oct-1, HSF-1, E-26 like protein 1 (ELK-1) and *Aspergillus nidulans* developmental regulatory gene (AbaA). The MatInspector program revealed three putative transcription factor binding sites; Oct-1, a human muscle-specific Mt binding site and a member of the TEA/ATTS DNA binding domain factors (TEAD1/TEF1). The Oct-1 binding site was identified by three independent search programs and was thus further investigated.

The consensus sequence of the human Oct-1 binding site is ATT(A/T)GCAT [30]–[34]. The putative Oct-1 binding sequence in the 17 bp region is ATTGCAT. Although this sequence differs by one nucleotide from the consensus Oct1 binding site sequence, it has been suggested that the missing nucleotide in the sequence within the 17 bp region is not a crucial base for the binding of Oct-1. Instead, the essential nucleotides for binding Oct1 are located at the 5′ end of the sequence [31].

**Figure 7:**
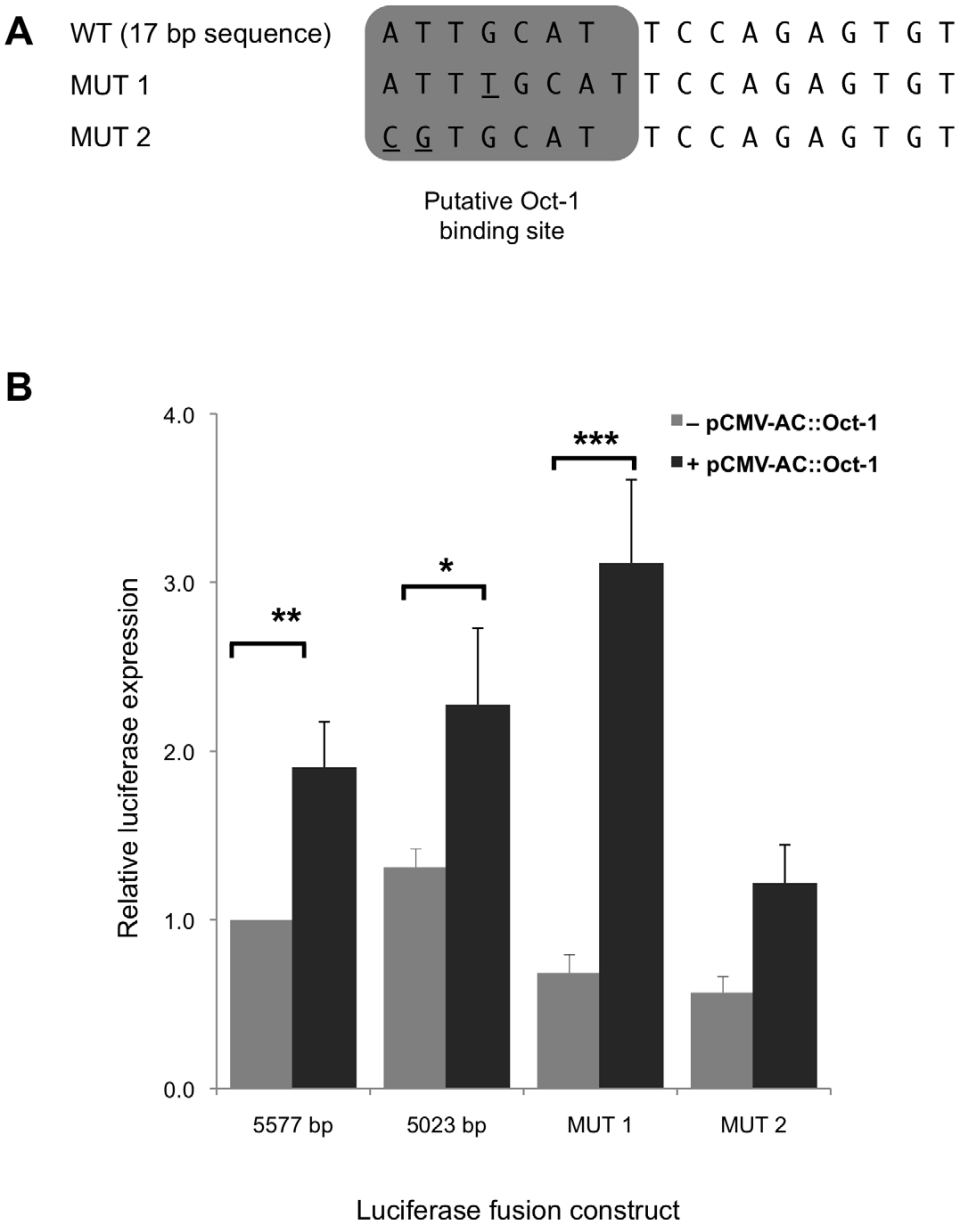
Regulatory effects of a putative Oct-1 binding site located within conserved region 1. (A) Two mutant constructs were generated by site directed mutagenesis within a putative Oct-1 binding site within the identified 17 bp nucleotide sequence. MUT 1 contains one additional extra base in the middle of the sequence and MUT 2 contains two bases substitution at the start of the sequence. (B) MUT 1 and MUT 2 were generated in the pGL3-Basic::5023 construct. Luciferase expression of the mutant constructs was analyzed following co-transfection of BE(2)-M17 cells with pGL3-Basic derivatives and Oct-1 transcription factor-encoding plasmid pCMV-AC::Oct-1. The overall levels of expression were then compared to the pGL3-Basic::5577 vector, the expression value of which was set as 1. Assays were performed in triplicate on at least three independent occasions. Error bars represent standard error of the mean. *p<0.05; **p<0.01; ***p<0.001.

A number of mutant constructs were generated by site directed mutagenesis. pGL3-Basic::5023 was utilized as the starting template for PCR amplification to create the mutant constructs. One derivative (designated MUT-1) was generated to contain an extra base to exactly match the consensus Oct-1 binding site. A second construct (MUT-2) was generated with two altered bases at the start of the sequence (Fig. 7A). Alteration of the two bases in MUT-2 was anticipated to inhibit Oct1 binding [31].

Transfection of each construct was performed in BE(2)-M17 cells. The expression of the pGL3-Basic::MUT1 and pGL3-Basic::MUT2 constructs was compared to the expression of pGL3-Basic::5577. Overexpression of the Oct-1 transcription factor was accomplished by the co-transfection of the pCMV-AC::Oct-1 construct that expresses human Oct-1 (Origene). As a negative control, a construct containing only the backbone plasmid pCMV-AC was generated by excising the *Oct-1* sequence from the original vector.

The overexpression of the Oct-1 transcription factor in the BE(2)-M17 cell line caused a marked increase in the levels of luciferase expression produced by the positive controls pGL3-Basic::5577 and pGL3-Basic::5023 as well as pGL3::MUT1 (*p* = 0.003, *p* = 0.05 and *p* = 0.009, respectively) (Fig. 7B). Oct-1 overexpression did not result in any significant change in the luciferase expression of the pGL3-Basic::MUT2 vector (Fig. 7B). These results indicated that the Oct-1 binding site had been disrupted in the pGL3-Basic::MUT2 construct and supported the previous findings that the first two nucleotides in the Oct-1 binding site are crucial for Oct1 binding [31]. This induction was also specific to the production of Oct-1 as there was no significant change in the expression of any construct in the presence of the negative control pCMV-AC vector (data not shown). There was also increased inducibility upon insertion of an extra base in pGL3-Basic::MUT1 (4.5 fold increase) compared to pGL3-Basic::5023 (1.7 fold increase) and pGL3-Basic::5023 (1.9 fold increase). This is also consistent with other reported studies in regards to the full Oct-1 binding site sequence [30]–[34].

Altogether, these results indicate that there is an Oct-1 binding site within the 17 bp region that is important in *FXN* gene expression and that the first two bases of the Oct1 binding site are critical for Oct-1 binding.

## Discussion

The rapid advances that have resulted from the sequencing of the human genome and those of other species, and the development of bioinformatics programs and matrices have facilitated the study of gene regulation. Prior to this study, information about the regulation of the normal human *FXN* gene was limited to the regions immediately flanking the first exon of the gene [7], [9], [16], [35]. There was no data on the position of any long-range, *cis*-acting regulatory sequences or of known transcription factors that may control *FXN* gene expression. In this study, *in silico* approaches were used in conjunction with different reporter systems to identify and better understand potential long distance *cis*-acting regulatory elements controlling the expression of the human *FXN* gene.

The search of regulatory elements in this study was restricted to the intergenic region upstream of exon 1 of the *FXN* gene to the *PRKACG* gene. A detailed analysis of this 21.3 kb region revealed the presence of eight highly conserved non-coding regions. The eight regions are evolutionarily conserved from human to mouse and some to even more divergent species. Each of the identified regions is within the size range of 40–750 bp and harbor between 40% to greater than 70% homology between species. The linear order of the identified conserved regions is maintained across the species analyzed. These findings make the eight regions strong candidates to contain key regulatory elements for *FXN* gene expression.

The eight identified conserved sequences were analyzed via their deletion in the context of the whole *FXN* locus within a dual-reporter BAC-based assay. BACs are very useful research tools as they can contain the entire intact locus of a gene, including surrounding regulatory elements, thus allowing the recapitulation of normal gene expression patterns. In addition, the positional information of most regulatory elements required for normal gene expression will be naturally preserved. In this approach, a reporter gene is fused to the coding sequence of the gene of interest in the context of its intact genomic locus and the whole construct is then used to study gene expression in transient transfection studies. An advantage of transient assays over the integration of BAC constructs into the host genome is the avoidance of variation produced by different integration sites, the influences of flanking genomic sequences, and in differences in transgene copy number at the site of integration.

The RP11-265B8 BAC clone utilized in this study contains the *FXN* gene and significant amounts of flanking sequences, thus making it likely to contain the intact *FXN* locus. This BAC construct has also been shown to rescue the embryonic lethality of the homozygous knockout of the mouse *Fxn* gene [29] indicating that most, if not all, regulatory elements required for normal *FXN* gene expression are present within this clone. This BAC clone was also used to develop the first genomic reporter for *FXN* expression, based on the targeted insertion of an EGFP cassette following the final codon of exon 5a [28]. The DsRed-Express reporter was introduced into the backbone of BAC clone to serve as an internal control for transfection efficiency and vector copy number. The presence of the two fluorescent markers enabled the monitoring of the effects of modifications of conserved regions and permitted correction for differing transfection efficiencies.

Modifications of each of the conserved non-coding regions identified *in silico* were carried out on the dual-reporter BAC construct. The deletion of conserved region 4 (9.5 kb upstream of exon 1) and the deletion of conserved region 5 (12.4 kb upstream of exon 1) resulted in an increase in *FXN* gene activity by 30% and 20%, respectively. It is therefore hypothesized that these regions contain silencer elements or binding sites for repressors, which normally serve to dampen *FXN* gene expression. Removal of these sites may therefore alleviate the repression of gene expression and result in higher levels of gene expression in individuals with FRDA.

In contrast, the deletion of conserved region 1 (5.3 kb upstream of exon 1) resulted in a significant reduction in *FXN* gene expression to 65% of the level of the unmodified gene. The deletion of conserved region 6, located 15.5 kb upstream of exon 1, produced a similar level of reduction in gene expression to 64% of the levels of the unmodified gene. These findings indicate that there are sequences within both of these conserved regions that play a role in the expression of the *FXN* gene. The deletion of conserved region 7 (16.5 kb upstream of exon 1) resulted in a more modest reduction in gene expression (75% of the levels of the unmodified gene). A possibility is that sequences in these conserved regions act as enhancers or binding sites for transcription factors that activate gene expression. In vertebrates, during transcription, a number of factors are assembled together encompassing the core promoter elements of the gene along with other *cis-*regulatory sequences which include enhancer elements. These enhancer elements could further recruit gene regulatory proteins such as transcription factors and further increase gene expression via enhancement of the overall rate of transcription. Deletion of these small regions in the assay resulted in the possible loss of enhancer effect and the loss of binding sites for certain transcription factors leading to an overall reduction in *FXN* gene expression.

To complement the BAC reporter assays, luciferase reporter assays were also used to study the potential role of each region. Unlike the BAC constructs, the luciferase reporter is present on a small, compact plasmid and is thus easier to manipulate. Candidate regulatory sequences were cloned adjacent to the reporter gene within a small construct. Subsequently, each construct was transfected into cultured cells to monitor gene expression.

Different sized fragments each containing the predicted *FXN* promoter region [7] and one or more of the conserved non-coding regions were amplified by PCR and cloned into the pGL3-Basic vector. The smallest DNA fragment cloned contained the 1,269 bp region immediately upstream of exon 1. This fragment included the promoter region of the *FXN* gene but did not contain any of the identified conserved non-coding regions. Greene and colleagues previously generated four constructs containing different regions upstream of the *FXN* gene, ranging from 1,255 bp to 4,356 bp. Their observation in a mouse myoblast cell line showed that no significant changes in luciferase expression was apparent between these four constructs leading to the conclusion that there might not be important regulatory elements located between positions 1,255 bp and 4,356 bp upstream of exon 1 [7]. Their results suggested that the minimal promoter activity is contained within the first 1,255 bp immediately upstream of exon 1. In order to replicate these findings, fragments containing 1,269 bp and 4,923 bp upstream of exon 1 were cloned into pGL3-Basic in this study. The other inserts gradually increased in size to successively include the identified conserved regions.

Each of the pGL3-Basic derived constructs were analyzed in two separate cell lines, HeLa and BE(2)-M17. The parental pGL3-Basic vector was used as the negative control while the luciferase expression of each construct was compared to the construct containing the 1,269 bp fragment that had been shown to contain the minimal *FXN* promoter. The expression patterns produced by the two smallest constructs containing the 1,269 bp and 4,923 bp regions upstream of exon 1 in HeLa cells were comparable to those reported by Greene and colleagues [7]. This was despite differences in the cell types and species of origin between the two studies. There was no significant difference in the relative luciferase activity of the two constructs in HeLa cells. However, in BE(2)-M17 cells there was a significant decrease in gene expression observed for the construct containing the 4,923 bp fragment compared to the minimal promoter fragment. The 4,923 bp fragment does not contain any of the identified conserved regions, but our results indicated that there is a region located between positions 1,269 bp and 4,923 bp upstream of the *FXN* gene that elicits an inhibitory effect on gene expression in this cell line.

A significant increase in *FXN* expression was observed in the construct containing the 5,577 bp and larger inserts in both HeLa and BE(2)-M17 cells indicating that there could be important regulatory elements harbored at least within the 5,577 bp fragment. Variations and fluctuations in the levels of expression between different constructs may be due to longer regions containing multiple regulatory elements which may enhance or diminish gene expression. There were also differences in the patterns of expression in the two cell lines indicating likely differences in the specific transcription factors responsible for modulating *FXN* expression in the two cell types.

The 5,577 bp fragment that elicited a marked increase in *FXN* gene expression includes conserved region 1. This observation complemented the findings from the BAC genomic reporter assays, where the absence of this conserved non-coding region resulted in a significant reduction in *FXN* gene expression. Both sets of data strongly suggested that this region contains an important regulatory element essential for maximal *FXN* gene expression.

In order to refine the location(s) of regulatory element(s) within this region, additional truncated fragments terminating in the region between 4,923 bp and 5,577 bp upstream of exon 1 were generated. The region of interest was narrowed down to a 17 bp sequence located between 4,944 bp and 4,961 bp upstream of exon 1 of the *FXN* gene. The removal of the 17 bp sequence resulted in a reduction of gene expression of 45% and 67% in HeLa and BE(2)-M17 cell lines, respectively. The 17 bp sequence is located within conserved non-coding region 1 and it is sufficient and necessary for maximal *FXN* gene expression.

This finding in the luciferase system was further supported by the genomic reporter assay system. Deletion of the 17 bp region in the context of the entire human *FXN* gene in the BAC reporter clearly demonstrated a reduction in EGFP expression, by flow cytometry analysis and fluorescent microscopy, when compared to the control dual reporter unmodified plasmid. Furthermore, the EGFP expression of this construct was comparable to that observed where the entire conserved region 1 was deleted (approximately 65% of the normal gene expression). It was therefore evident that the absence of this region in the BAC genomic reporter assay resulted in a decrease in *FXN* gene expression and the presence of this region in the small plasmid reporter system resulted in an increase in gene expression.

In an attempt to comprehensively identify regulatory element(s) located within the 17 bp sequence, *in silico* approaches were undertaken. A binding site for the Oct-1 transcription factor was the most likely candidate identified by three different search tools.

Oct-1 is known to bind to an 8-bp sequence termed an octamer motif. Mice lacking Oct-1 die during early development indicating that the presence of this transcription factor is essential for life [36]. Unlike other members of the POU (pituitary-specific Pit-1, octamer-binding proteins Oct-1 and Oct-2, and neural Unc-86) protein family, this transcription factor is expressed ubiquitously and no specific temporal or spatial pattern is observed [37]. Oct-1 is expressed in all eukaryotic cells and regulates, either positively or negatively, the expression of a variety of genes [38]. This ability is attributed to its flexibility in binding DNA as a monomer, homodimer, or heterodimer [39]. This regulatory factor has also been shown to be important for tissue and cell-specific transcription as well as the transcription of a number of housekeeping genes. Oct-1 has also been shown to participate in recruiting pre-initiation complexes in the promoter region of genes lacking a TATA box by functionally substituting the role of transcription binding protein (TBP) [40], [41]. In the case of the *FXN* gene, this finding is very relevant as there is no TATA box sequence located within the vicinity of the *FXN* promoter region [7].

The candidate Oct-1 sequence in the 17 bp region (ATTGCAT) did not exactly match the human Oct-1 binding site consensus sequence (ATT(A/T)GCAT) [30]–[34]. The missing nucleotide has been found not to be critical for Oct-1 binding [31]. It has been found that the essential nucleotides for Oct-1 binding are located at the 5′ end of the sequence [31]. It is well accepted that the sequence of transcription factor binding sites can often differ in different parts of the genome.

Two constructs were generated by site directed mutagenesis. The first construct had one base pair added to match the consensus human Oct-1 binding site and the second had a two base pair alteration at the start of the sequence [31]. Upon Oct-1 transcription factor overexpression, significant increases in luciferase expression were seen to be greatest with the construct containing the one base pair addition as well as controls that contained the 17 bp region. This increase was however not seen with the construct containing the two base pairs modification (pGL3-Basic::MUT2). In line with a previous study [31], substitution of the first two base pairs resulted in the loss of Oct-1 transcription factor inducibility.

Altogether, this result strongly suggested that the 17 bp region delineated from conserved region 1 contains an Oct-1 binding site and that the substitution of the first two bases within the consensus sequence disrupted this binding. This indicates that the crucial nucleotides for the transcription factor binding are located at the start of the Oct-1 sequence. Furthermore, it was also shown that the presence of an additional base within the pGL3-Basic::MUT1 construct to match the consensus Oct-1 binding site indeed increased the luciferase reporter expression. The presence of this extra sequence may strengthen Oct-1 transcription factor binding in the presence of abundant transcription factor.

The findings of the current study are important and may influence future clinical therapeutic strategies for FRDA. It is hypothesized that any increase in frataxin levels should prove beneficial, while a several-fold increase could be sufficient to halt disease progression. Induction of *FXN* gene expression directly addresses the primary issue of frataxin deficiency. Therapies for hemoglobinopathies have been developed based on knowledge of gene regulation. In β-thalassemia, where there is a disruption of β-globin production, increasing γ-globin gene expression ameliorates disease severity [15]. As Oct-1 reduces the transcription of γ-globin, a decrease in Oct-1 binding results in an increase in γ-globin gene expression. In one study, decoy oligonucleotides containing the Oct-1 consensus sequence were able to compete with the endogenous γ-globin *cis-*regulatory elements *in vitro* leading to the removal of the transcription factor from the endogenous targets and thus altering transcription of the target gene [42]. Although this is an example of the removal a transcription factor in order to acquire a higher level of gene expression, it does give an insight into the possibility in modulating the level of the Oct-1 transcription factor to influence *FXN* gene expression as a potential clinical therapy for FRDA.

In conclusion, we have demonstrated that highly conserved regions upstream of the *FXN* gene influence gene expression. We have identified an Oct-1 binding site about 4.95 kb from the start codon of the *FXN* gene that is necessary for normal gene expression and whose deletion results in decreased gene expression.

## Materials and Methods

### Bioinformatic analyses

Comparative and regulatory tracks available on the UCSC Genome Browser [18], [19] were examined in combination with other external data to find regions of interest in common between different sets of data. External data consisted of putative cis-regulatory regions including common transcription factor binding sites using BlastZ [27].

### Bacterial strains and oligonucleotides

*Escherichia coli* strains used were DH10B (Invitrogen, Carlsbad, CA) and its derivatives DY380 and EL250 [43]. All were grown in Luria-Bertani (LB) liquid culture or on LB agar at 37°C for DH10B and at 30°C for DY380 and EL250. Media were supplemented with appropriate antibiotics. Sequences of oligonucleotides used in this study are presented in Table S1.

### Site directed mutagenesis

Site directed mutagenesis was performed using the QuikChange Site-Directed Mutagenesis Kit (Agilent Technology, Forest Hill, Australia) according to the manufacturer's instructions.

### Homologous recombination

Insertion of linear PCR-generated fragments into BAC DNA was carried out by inducible homologous recombination (recombineering) as previously described [43]. *E. coli* DY380 cells containing a BAC vector were cultured in LB containing appropriate antibiotics at 30°C to an OD_600_ of 0.45. Expression of the *exo*, *bet* and *gam* genes was induced by incubation at 42°C for 20 min. Cells were harvested and prepared for electroporation, and then 0.5–2.0 µg of purified PCR product was electroporated into electrocompetent cells. Following incubation at 30°C in 1 ml of LB for 3 h, cells were plated on LB agar containing appropriate antibiotics and colonies allowed to form over a 24–48 h period.

### Construction of a dual reporter bacterial artificial chromosome

Homologous recombination was used to introduce a pCMVIE—DsRed-Express—ampicillin-resistance cassette into the backbone of the RP11-265B8::Ex5aEK BAC construct. PCR primers EXT-BAC265-DSRED-F and EXT-DSRED-R-ADDITION were used to amplify a 1.5 kb PCR product containing the DsRed-Express gene and CMV promoter (pCMVIE) from the pDsRed-Express-N1 plasmid (Clontech, Palo Alto, CA). The 21 nucleotides (nt) at the 3′ end of EXT-BAC265-DSRED-F anneal to a unique sequence on the pDsRed-Express-N1 plasmid and the remaining 21 nt correspond to a sequence upstream of the chloramphenicol-resistance gene on the RP11-265B8::Ex5aEK BAC backbone. The 21 nt at the 3′ end of EXT-DSRED-R-ADDITION anneal to a unique sequence on pDsRed-Express-N1 and the remaining 23 nt are additional bases to facilitate the second stage of the fusion (stitching) PCR process [44]. PCR primers EXT-AMP-B-R-BAC265 and EXT-ADDITION-AMP-B-F were used to amplify a 1.1 kb fragment containing the ampicillin-resistance gene from the pBR322 plasmid (New England Biolabs, Ipswich, MA). The 21 nt at the 3′ end of EXT-AMP-B-R-BAC265 anneal to a unique sequence on pBR322 downstream of the ampicillin-resistance gene and the remaining 23 nt correspond to a sequence downstream of the chloramphenicol-resistance gene on the RP11-265B8::Ex5aEK BAC backbone. The 22 nt at the 3′ end of the EXT-ADDITION-AMP-B-F primer anneal to a unique sequence on pBR322 between the ampicillin-resistance gene and the tetracycline-resistance gene and the remaining 21 nt are additional bases to facilitate the fusion PCR process. PCR products were recovered from agarose gels and purified using a QIAquick Gel Extraction Kit (Qiagen, Doncaster, Victoria, Australia) according to the manufacturer's instructions.

The two PCR products were fused into a single DNA fragment by fusion PCR utilizing the introduced overlapping sequence and primers EXT-BAC-DsRed-F and EXT-AMP-B-R-BAC265. The sequence of the 2.6 kb fragment was verified by DNA sequencing performed using Big Dye V3.1 Cycle Sequencing kit (Applied Biosystems, Victoria, Australia) according to the manufacturer's recommendations.

A targeting cassette containing the pCMVIE–DsRed–ampicillin-resistance sequence and incorporating homology arms for the recombination was prepared by PCR performed with primers EXT-TGT-R-DSAMP and EXT-TGT-F-DSAMP. The first 49 nt of the EXT-TGT-F-DSAMP primer correspond to a sequence upstream of the chloramphenicol-resistance gene on the RP11-265B8::Ex5aEK BAC backbone and the 21 nt at the 3′ end of the primer anneal to a sequence at the 5′ end of the pCMVIE—DsRed-Express—ampicillin-resistance PCR product. The first 47 nt of the EXT-TGT-R-DSAMP primer correspond to a sequence downstream of the chloramphenicol-resistance gene on the RP11-265B8::Ex5aEK BAC backbone and the 23 nt at the 3′ end of the primer anneal to a sequence at the 3′ end of the pCMVIE—DsRed-Express—ampicillin-resistance PCR product.

Following the insertion of the pCMVIE—DsRed-Express—ampicillin-resistance cassette into the RP11-265B8::Ex5aEK by homologous recombination, the integrity of the modified construct, termed RP11-265B8::Ex5a-EK-DsAmp (Dual Reporter), was verified by PCR, restriction digestion and DNA sequencing.

### Targeted deletions in the dual reporter BAC construct

A cassette consisting of the chloramphenicol-resistance gene flanked by FRT sites was generated by PCR using primers FRT-CAM-F and FRT-CAM-R. FRT-CAM-F contains an *Eco*RI site, 35 nt corresponding to an FRT sequence and 23 nt that anneal to a region upstream of the chloramphenicol-resistance gene in pBACe3.6 [45]. FRT-CAM-R contains a *Bam*HI site, an FRT site and 24 nt homologous to a region downstream of the chloramphenicol-resistance gene. The PCR product was digested with *Eco*RI and *Bam*HI and cloned into pUC19. The resulting construct was designated pUC-FRT–CAM and was used to amplify specific targeted deletion cassettes containing the FRT–chloramphenicol-resistance–FRT sequence. The 50 nt at the 5′ end of each primer represent the homology targeting sequences in the *FXN* locus and the remainder are those used to prime amplification of the FRT–chloramphenicol-resistance–FRT sequence. The modular structure of the PCR-amplified targeting cassette can be represented as:[Upstream homology block]–FRT–[chloramphenicol-resistance gene]–FRT–[Downstream homology block].

Targeted deletions were performed via induced homologous recombination in DY380 cells containing the RP11-265B8::Ex5a-EK-DsAmp (Dual Reporter) construct. The removal of the chloramphenicol-resistance gene was achieved by induction of Flp recombinase following the transformation of each construct into EL250 cells. The integrity of each modified construct was verified by PCR, restriction digestion and DNA sequencing.

### Construction of luciferase reporter constructs

A set of progressively longer, nested DNA fragments containing the endogenous *FXN* promoter and one or multiple conserved non-coding regions was amplified by PCR. A common reverse PCR primer (FXN-R-F) that annealed to the region just upstream of the *FXN* start codon and incorporating a *Hin*dIII site was used in all the PCR reactions. Forward primers incorporating a *Bgl*II site were designed to anneal to different locations upstream of the *FXN* gene. The RP11-265B8 BAC clone was used as a template for PCR-amplification using the Expand Long Template PCR kit (Roche Applied Science, Castle Hill, NSW, Australia). PCR products were gel extracted, digested with *Hin*dIII and *Bgl*II and cloned into the same sites of the pGL3-Basic vector (Promega, Alexandria, NSW, Australia).

As it was not possible to amplify fragments larger than 9 kb in sufficient quantity for cloning, an alternative strategy taking advantage of a naturally occurring *Pml*I site 8.8 kb upstream of the *FXN* gene was used. A reverse primer incorporating a *Pml*I site was used in conjunction with forward primers incorporating a *Bgl*II site. PCR fragments were cloned into a pGL3-Basic-derived construct into which a 8,914 bp fragment had been cloned (Fig. 3) following digestion with *Pml*I and *Bgl*II.

### Tissue culture maintenance, transfection and analysis of BAC and luciferase constructs

BHK-21 and HeLa cells were maintained in Dulbecco's modified Eagle's medium (DMEM) (Sigma, Sydney, NSW, Australia). BE(2)-M17 cells were maintained in GIBCO-OptiMEM (Invitrogen, Carlsbad, CA). All media were supplemented with 10% fetal calf serum (FCS), 100 U/ml penicillin and 100 µg/ml streptomycin. Cell lines were obtained from the ATCC.

For BAC transfection procedures, BHK-21 cells were plated at a density of 2×10^5^ cells per well in 6-well culture plates and allowed to reach 80% confluency. Cells were transfected with 20 µg of purified DNA using LipofectAMINE (Invitrogen, Carlsbad, CA), at a 4∶1 lipid to DNA ratio, according to the manufacturer's protocol. Analysis of the cells was performed 72 hours post-transfection. Cells to be analyzed were trypsinized, collected in 4 ml of DMEM and resuspended in 0.5 ml of clear phosphate buffered saline (PBS) containing 1% FCS. Flow cytometry was performed on an LSR II system (BD Biosciences, San Jose, CA). Data was analyzed with FACSDiVa software (BD Biosciences). Transfection of each BAC construct and flow cytometric measurement of fluorescent reporter expression were conducted in triplicate on three separate occasions.

Expression of EGFP from each deletion construct was compared to the unmodified RP11-265B8::Ex5a-EK-DsAmp (Dual Reporter). DsRed-Express expression was used as an internal control to normalize for transfection efficiency and vector number copy. In each case, the mean peak fluorescence (MPF) level of EGFP representing *FXN* gene expression produced by each construct was normalized against the MPF of Ds-Red-Express. The normalized expression of each construct was then compared to the normalized value of the unmodified RP11-265B8::Ex5a-EK-DsAmp (Dual Reporter) which was set as 1.

For the luciferase transfection procedures, HeLa cells were plated at a density of 2.5×10^4^ cells per well in 24-well culture plates and allowed to reach 80% confluency. Cells were transfected with purified DNA using Fugene HD (Roche, Basel, Switzerland), at a 3.5∶1 lipid to DNA ratio, according to the manufacturer's protocol.

BE(2)-M17 cells were plated at a density of 2.5×10^4^ cells per well in 48-well culture plates and allowed to reach 80% confluency. Cells were transfected with purified DNA using DMRIE-C (Invitrogen, Carlsbad, CA), at a 4∶1 lipid to DNA ratio, according to the manufacturer's protocol.

Each construct containing a firefly luciferase reporter was transfected together with the control plasmid, pRL-SV40 that expresses *Renilla* luciferase and acted as a transfection control. The unmodified pGL3-Basic construct was included in the assay as a negative control. Equimolar amounts of each DNA construct were transfected taking into account the differences in size between the constructs.

Transfection of each construct was performed in triplicate on at least three separate occasions in both cell lines. The chemiluminescent expression from both luciferase vectors was analyzed 48-hours post transfection using the Dual-Glo Luciferase Assay system (Promega). Cells were prepared according to the manufacturer's protocol. The firefly and *Renilla* expression were analyzed in a FLUOstar OPTIMA microplate reader (BMG Labtech, Cary, NC). The expression of the firefly luciferase reporter on the pGL3-Basic derivative constructs was normalized by comparison to *Renilla* luciferase expression from the pRL-SV40 control vector. The expression of all pGL3-Basic derivative constructs was then compared to the expression of the smallest construct containing the 1,269 bp fragment (pGL3-Basic::1269) which was set as 1.

#### Statistical analyses

For all transfection assays, the experimental data is reported as the mean ± the standard error of the mean (S.E.M) of the triplicate assays in at least three independent experiments. Comparisons were made between groups of equal size by Student's t-tests for paired data using Excel (Microsoft, Redmond, WA). Data were considered significantly different at *p*<0.05.

#### Transcription factor binding site programs

The transcription factor binding site searches were performed using the Alibaba 2.1, Match 1.0 (http://www.gene-regulation.com/pub/programs.html) and MatInspector programs (http://www.genomatix.de/online_help/help_matinspector/matinspector_help.html).

## Supporting Information

Figure S1 **Fluorescence microscopy imaging of cells containing BAC dual-reporter deletion constructs.** BHK-21 cells were transfected with the RP11-265B8::Ex5a-EK-DsAmp (Dual Reporter) construct and derivatives individually containing deletions of the identified conserved non-coding regions. Imaging was performed 72 hours post transfection. Left image of each panel shows red fluorescence corresponding to Ds-Red Express expression. Right image of each panel shows green fluorescence corresponding to *FXN* gene expression.
(TIF)

Figure S2 **Fluorescence microscopy imaging of cells containing the BAC dual-reporter with deletions in conserved region 1.** BHK-21 cells were transfected with the RP11-265B8::Ex5a-EK-DsAmp (Dual Reporter) construct and derivatives individually containing deletions of the entire conserved region 1 or the identified 17 bp sequence within conserved region 1. Imaging was 72 hours post transfection. Left image of each panel obtained with transmitted light. Middle image of each panel shows red fluorescence corresponding to Ds-Red Express expression. Right image of each panel shows green fluorescence corresponding to *FXN* gene expression.
(TIF)

Table S1 **Lists of oligonucleotides used in this study.** Nucleotides shown in plain text: part of the oligonucleotide directed to prime amplification; Underlined nucleotides; part of homology targeting arms; Double underlined nucleotides; FRT sequences; Underlined and italicized nucleotides: restriction enzyme sites.
(DOC)
