# Supplementary figures and images for: Long Range Regulation of Human FXN Gene Expression
Source: PLoS One. 2011 Jul 8;6(7):e22001. doi: 10.1371/journal.pone.0022001 (PMC3132285; doi:10.1371/journal.pone.0022001)

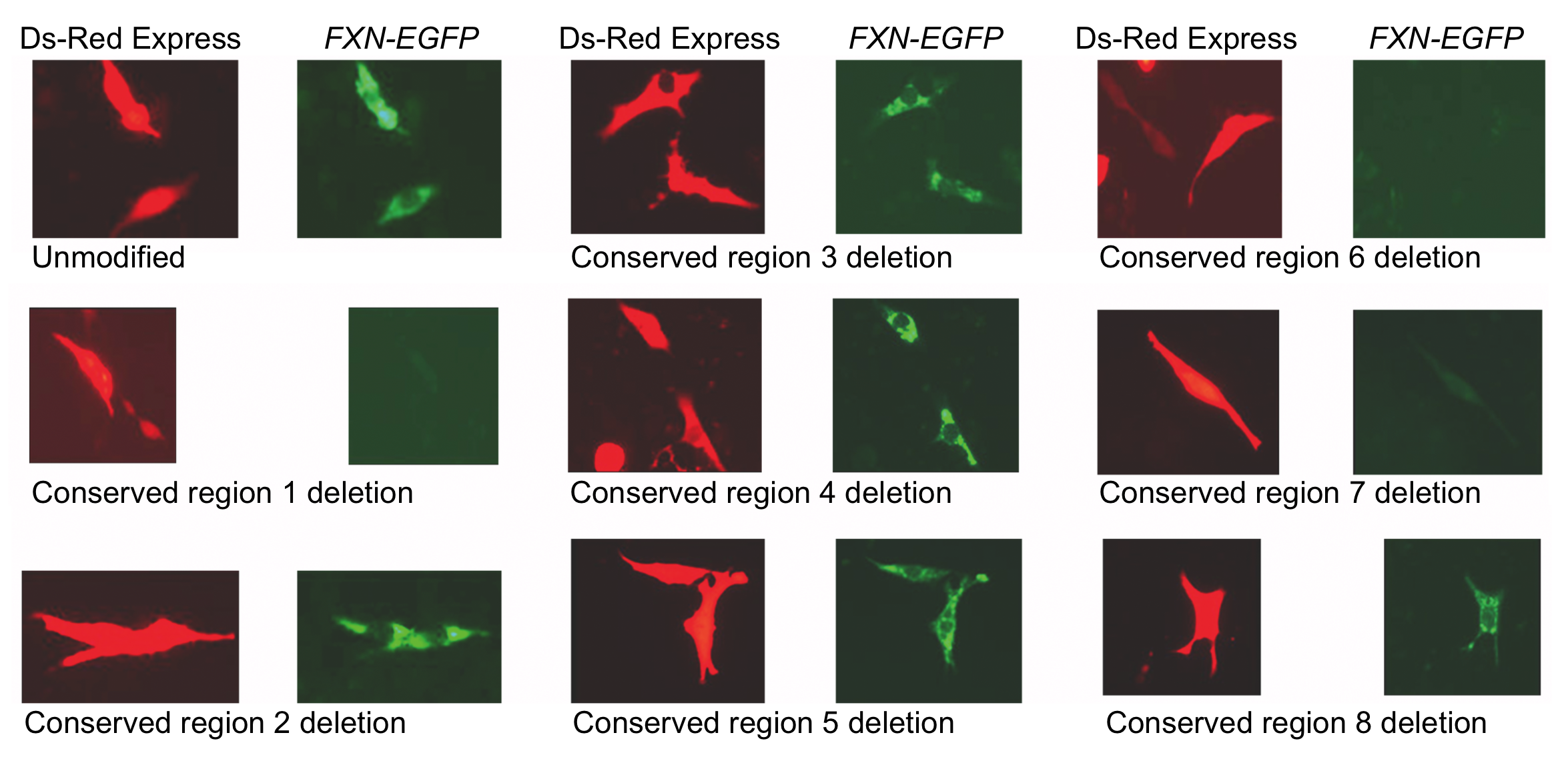

Supplement: Figure S1 — Fluorescence microscopy imaging of cells containing BAC dual-reporter deletion constructs. BHK-21 cells were transfected with the RP11-265B8::Ex5a-EK-DsAmp (Dual Reporter) construct and derivatives individually containing deletions of the identified conserved non-coding regions. Imaging was performed 72 hours post transfection. Left image of each panel shows red fluorescence corresponding to Ds-Red Express expression. Right image of each panel shows green fluorescence corresponding to FXN gene expression. (TIF) [file pone.0022001.s001.tif]

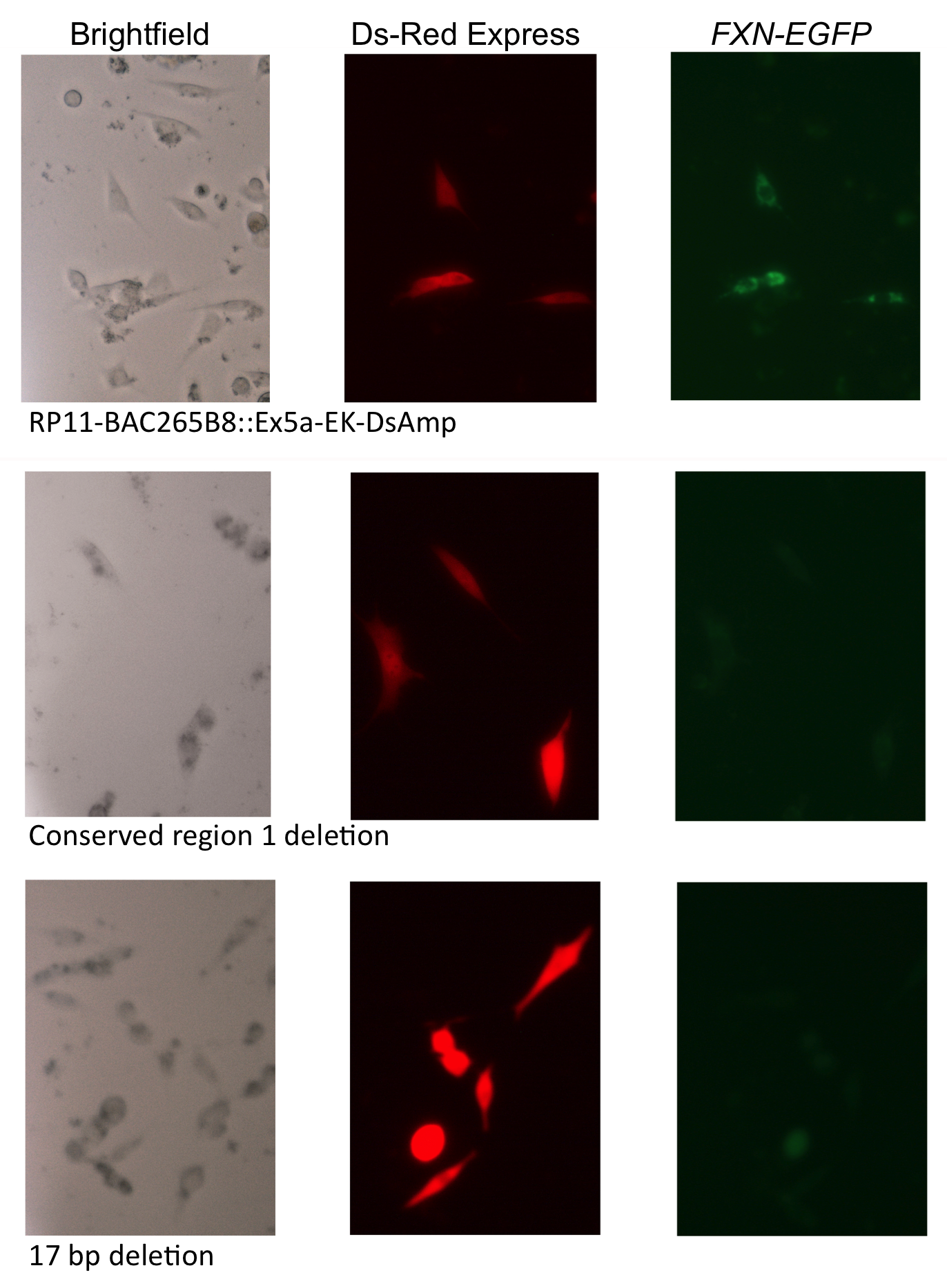

Supplement: Figure S2 — Fluorescence microscopy imaging of cells containing the BAC dual-reporter with deletions in conserved region 1. BHK-21 cells were transfected with the RP11-265B8::Ex5a-EK-DsAmp (Dual Reporter) construct and derivatives individually containing deletions of the entire conserved region 1 or the identified 17 bp sequence within conserved region 1. Imaging was 72 hours post transfection. Left image of each panel obtained with transmitted light. Middle image of each panel shows red fluorescence corresponding to Ds-Red Express expression. Right image of each panel shows green fluorescence corresponding to FXN gene expression. (TIF) [file pone.0022001.s002.tif]
