# Supplementary material for: Long Range Regulation of Human FXN Gene Expression
Source: PLoS One. 2011 Jul 8;6(7):e22001. doi: 10.1371/journal.pone.0022001 (PMC3132285; doi:10.1371/journal.pone.0022001)
Supplement: Table S1 — Lists of oligonucleotides used in this study. Nucleotides shown in plain text: part of the oligonucleotide directed to prime amplification; Underlined nucleotides; part of homology targeting arms; Double underlined nucleotides; FRT sequences; Underlined and italicized nucleotides: restriction enzyme sites. (DOC) [file pone.0022001.s003.doc]

**Table S1**. Lists of oligonucleotides used in this study.

| Name | Sequence (5’ → 3’) |
| --- | --- |
| **Primers for creating BAC dual luciferase construct** | |
| EGFP-Probe-F | ATGGTGAGCAAGGGCGAGGAGCTGTT |
| EGFP-Probe-R | CTGGGTGCTCAGGTAGTGGTTGTC |
| pBR-Tet-F | TCTAGTTGTGGTTTGTCCAAACTCATCAATGTATCTTAAGGCGTAAATTGGTATCACGAGGCCCTTTCGTCT |
| pBR-Tet-R | TATCGACAGAGTGCCAGCCCTGGGACCGAACCCCGCGTTTATGAACAAACCCATTCATGTTGTTGCTCAGGT |
| DsRed-Tet-B-R | GCCACATCCAGCGCAAAAACCTTCGTGTAGACTTCCGTTGAACTGATGGACCCTGATTCTGTGGATAACCGTAT |
| DsRed-Tet-B-F | cttattcaggcgtagcaaccaggcgtttaagggcaccaataactgccttattgcagcagcagtcgcttcacgt |
| FRT-CAM-R | *TTTGGATCC*GAAGTTCCTATTCTCTAGGAAAGTATAGGAACTTCACTCATGTTTGACAGCTTATCATC |
| Amp-Forward | gaatgtatttagaaaaataaaca |
| Amp-Reverse | tctcatgaccaaaatcccttaac |
| EXT-BAC265-DSRED-F | CCGGGAAGCCCTGGGCCAACTATGCATTAGTTATTAATAGTA |
| EXT-DSRED-R-ADDITION | CCTATGATCAATCCTCAGCATAGTTTGGACAAACCACAACTAGA |
| EXT-ADDITION-AMP-B-F | ATGCTGAGGATTGATCATAGGATGATAATAATGGTTTCTTAGA |
| EXT-AMP-B-R-BAC265 | GTTTAAGGGCACCAATAACTGCCGTTAAGGGATTTTGGTCATGAGA |
| DsRed into CmR-F | AGTTGGCCCAGGGCTTCCCGGTATCAACAGGGACACCAGGATTTATTTATTTGCAGCAGCAGTCGCTTCACGT |
| DsRed into CmR-R | ggcagttattggtgcccttaaacgcctggttgctacgcctgaataagtgTTTTGTGATGCTCGTCAGGGG |
| Ext-TGT- F-DsAMP | acggaagatcacttcgcagaataaataaatcctggtgtccctgttgataccgggaagccctgggccaact |
| **Primers for creating luciferase constructs** | |
| 1269 FXN-F | tttt*AGATCT*ACCACACTCGGCTCACATTTGACA |
| 4923 FXN-F | tttt*AGATCT*ATCAGTCTCCTGGGACAAACAGCTG |
| 4944 fxn-f | TTTT*AGATCT*ggatttgactctagtgccat |
| 4961 FXN-F | TTTT*AGATCT*ATTGCATTCCAGAGTGTGGATTTG |
| 5023 FXN-F | TTTT*AGATCT*TTGATTACTTTGCTGAGATC |
| 5123 FXN-F | TTTT*AGATCT*AATAAATCCAAACCTCCTTTTTTCT |
| 5443 FXN-F | TTTT*AGATCT*GCCAGCCTATGAGACTAGAAGCAAG |
| 6246 FXN-F | tttt*AGATCT*aacacacttcagtacctaaaactc |
| 7522 FXN-F | tttt*AGATCT*AGCACAGTGTGACTATGCTCGACT |
| 8914 FXN-F | tttt*AGATCT*aagccacaacctattttaacaacac |
| 11344 FXN-F | tttt*AGATCT*taatagtaccctcagtaaggctatt |
| 13629 FXN-F | tttt*AGATCT*TTTCTTTTGTGGGGGAGAATTTAC |
| 15814 FXN-F | tttt*AGATCT*cacctatgtaagcattttctctgca |
| 17077 FXN-F | TTTT*AGATCT*GAGGATGGGGGTAGGAATGT |
| FXN-R-F | tttt*aagctt*GCTGCTCCGGGTCTGCCGCCC |
| Pml-R | tggtaccagaatatagggtcagg |
| **Primers for creating BAC deletion constructs** | |
| 20kb-DEL-F | tcaacaatatggataaatccaaaaggcattatgcaagtgagagaagccaactgaagttcctatactttctagagaataggaacttcacctgtgacgg |
| 20kb-DEL-R | cccccacctgtgcaacaaagaactatccagtccaaaattctgccaagattCGAAGTTCCTATTCTCTAGAAAGTATAGGAACTTCACTCATGTTTG |
| 21kb-DEL-R | gagcagctagaggttagacctcaggaagaacttcccagcttagcactattCGAAGTTCCTATTCTCTAGAAAGTATAGGAACTTCACTCATGTTTG |
| region-1-del-f | CTGTCATAACCTTTACAAAGGTGGTTTCATTAGGCATATTAATGCAAATTctgaagttcctatactttctagagaataggaacttcacctgtgacgg |
| region-1-del-r | gctgtttgtcccaggagactgattatggcactagagtcaaatccacactcCGAAGTTCCTATTCTCTAGAAAGTATAGGAACTTCACTCATGTTTG |
| region-2-del-f | ttctcattttataaagacaccactcctttcacattaggacctacacttgactgaagttcctatactttctagagaataggaacttcacctgtgacgg |
| region-2-del-r | ttctcccaaagtccataggttgaaattttaactgccaggacactcagaatCGAAGTTCCTATTCTCTAGAAAGTATAGGAACTTCACTCATGTTTG |
| region-3-del-F | aaagtggccccggggtgccctctgtgaccacaggtgtataaacatgcctcctgaagttcctatactttctagagaataggaacttcacctgtgacgg |
| region-3-del-r | tatagtaggctagaccatctagctttgtgtaagtacactctatgatgttcCGAAGTTCCTATTCTCTAGAAAGTATAGGAACTTCACTCATGTTTG |
| region-4-del-F | TCCTTTCCCTGCAACCAGTGACTCTCCACTATGCAACAGTGTCAGGCTCActgaagttcctatactttctagagaataggaacttcacctgtgacgg |
| region-4-del-r | tgggggcctgggactctggaaagggttattttggggtttcagagtgattcCGAAGTTCCTATTCTCTAGAAAGTATAGGAACTTCACTCATGTTTG |
| region-5-del-f | aatggaagaaaggacctgaggttaaggaactggagattgtaatcagaaagctgaagttcctatactttctagagaataggaacttcacctgtgacgg |
| region-5-del-r | GGGTTGCTTGTTTGCCTATTTGCTGTTACATCCATTCTCTTCTCCTCATCCGAAGTTCCTATTCTCTAGAAAGTATAGGAACTTCACTCATGTTTG |
| region-6-del-f | CAGCATTTGGGTGCATGCCCTTGATTTAAATCGATTAATGTTTTCCCAGActgaagttcctatactttctagagaataggaacttcacctgtgacgg |
| region-6-del-r | taccccccagcaggaactgactccgcacaagaggacacctttgattccctCGAAGTTCCTATTCTCTAGAAAGTATAGGAACTTCACTCATGTTTG |
| region-7-del-f | AAAGCAGTGGTTCTTAATTTTAACTGCACATTGAAATCACCTGAGACATTctgaagttcctatactttctagagaataggaacttcacctgtgacgg |
| region-7-del-r | tttaaaagtcccaacgttcaagtcacatgcctgggcagttaagtcaatctCGAAGTTCCTATTCTCTAGAAAGTATAGGAACTTCACTCATGTTTG |
| region-8-del-f | GTTTCTACAGCTGTTGGCCAATAAGTTCCTTGCCTTCTGTCCTGAGCCCTctgaagttcctatactttctagagaataggaacttcacctgtgacgg |
| region-8-del-r | tctgtgctgccagtgcttatctgtctactaagttcaatcccagatttccaCGAAGTTCCTATTCTCTAGAAAGTATAGGAACTTCACTCATGTTTG |

Nucleotides shown in plain text: part of the oligonucleotide directed to prime amplification; Underlined nucleotides; part of homology targeting arms; Double underlined nucleotides; FRT sequences; Underlined and italicized nucleotides: restriction enzyme sites.
